# Comprehensive mouse microbiota genome catalog reveals major difference to its human counterpart

**DOI:** 10.1371/journal.pcbi.1009947

**Published:** 2022-03-08

**Authors:** Silas Kieser, Evgeny M. Zdobnov, Mirko Trajkovski

**Affiliations:** 1 Department of Cell Physiology and Metabolism, Faculty of Medicine, University of Geneva, Centre Medical Universitaire, Geneva, Switzerland; 2 Diabetes Center, Faculty of Medicine, University of Geneva, Geneva, Switzerland; 3 Swiss Institute of Bioinformatics, Geneva, Switzerland; 4 Department of Genetic Medicine and Development, Faculty of Medicine, University of Geneva, Centre Medical Universitaire, Geneva, Switzerland; 5 Institute of Genetics and Genomics in Geneva (iGE3), University of Geneva, Geneva, Switzerland; Genome Institute of Singapore, SINGAPORE

## Abstract

Mouse is the most used model for studying the impact of microbiota on its host, but the repertoire of species from the mouse gut microbiome remains largely unknown. Accordingly, the similarity between human and mouse microbiomes at a low taxonomic level is not clear. We construct a comprehensive mouse microbiota genome (CMMG) catalog by assembling all currently available mouse gut metagenomes and combining them with published reference and metagenome-assembled genomes. The 41’798 genomes cluster into 1’573 species, of which 78.1% are uncultured, and we discovered 226 new genera, seven new families, and one new order. CMMG enables an unprecedented coverage of the mouse gut microbiome exceeding 86%, increases the mapping rate over four-fold, and allows functional microbiota analyses of human and mouse linking them to the driver species. Comparing CMMG to microbiota from the unified human gastrointestinal genomes shows an overlap of 62% at the genus but only 10% at the species level, demonstrating that human and mouse gut microbiota are largely distinct. CMMG contains the most comprehensive collection of consistently functionally annotated species of the mouse and human microbiome to date, setting the ground for analysis of new and reanalysis of existing datasets at an unprecedented depth.

## Author summary

The microbiome plays an indispensable role in our health. Metagenomics enables valuable insights into the composition and functional potential of microbial populations. The analysis of metagenomic data is complex and depends on the availability of reference genomes. The mouse is the most used model for studying the impact of microbiota on its host. However, the microbial species living in the mouse gut remain poorly characterized. We created a comprehensive catalog of all bacterial species commonly living in the gut of laboratory mice by analyzing all publicly available metagenomes from the mouse gut. We collected almost 42 thousand bacterial genomes from 1’573 species, of which 78.1% are uncultured. Our catalog effectively answers the need for a genome reference for this microbiome and allows efficient analysis of mouse gut metagenomes down to the species level. We discovered that mice and humans harbor a largely distinct set of species in their gastrointestinal tracts, a hereto unfeasible analysis.

## Introduction

Mouse is the most used model for studying the microbiota importance due to several factors: availability of samples from different parts of the gastrointestinal tract, treatment options, controlled housing environment and diet, defined genetic background, and ethical considerations. However, the mouse gut microbiota has been poorly characterized. Most mouse microbiome studies are performed by sequencing 16S variable regions, sometimes mislabeled as metagenomics. While this technique has allowed a general overview of the microbiota bacterial taxonomic diversity down to the genus level, it is not suited for identifying species for most organisms [1]. Different species from the same genus and even subspecies from the same species can exert distinct functions [2], stressing the importance of annotating the gene content at the lowest taxonomic level. Shotgun metagenomics allows studying the full microbiota diversity of an environment, including uncultured microorganisms, viruses, and plasmids. But its interpretation is limited by the availability of reference genomes. Previous efforts led to the creation of a gene catalog of the mouse metagenome (MGC v1) [3], by sequencing fecal samples from mice with different genotypes and housed in different conditions. This catalog enables projecting known functional annotations of genes and allows up to 50% mapping rate of fecal shotgun sequences. However, the mapping rate of sequences from cecum samples is only 37%, and even an updated and extended version of the gene catalog [4] does not contain genomic references. Recent progress in the assembly of genomes from metagenomes led to a recovery of new species from the human gut and other environments [5–9]. The integrated mouse gut metagenomic catalog (iMGMC) [10] increased the fraction of reads mapped to genes compared to the MGC v1. However, mapping to the recovered metagenome-assembled genomes (MAGs) remained about 40% [10]. Lesker *et al*. also generated a set of 13,619 mouse-specific MAGs (mMAG) not integrated into the iMGMC, which was made available for further studies.

Here we report the creation of the Comprehensive mouse microbiota genome (CMMG) collection, achieved by assembling gut microbiomes sequenced by us and all publicly available mouse metagenomes. This resource improves the mapping rate of genomic reads from mouse fecal and cecum metagenomes to 86.2%, provides full classification down to species level, and enables uncovering compelling functional insights linking them to the driver species. This nearly complete catalog of the mouse gut bacterial species allows comparison between the newly assembled mouse gut microbiomes to the human counterpart, uncovering that human and mouse gut microbiota are largely distinct.

## Results

### Assembly of high-quality genomes from mouse gut metagenomes

**Fig 1:**
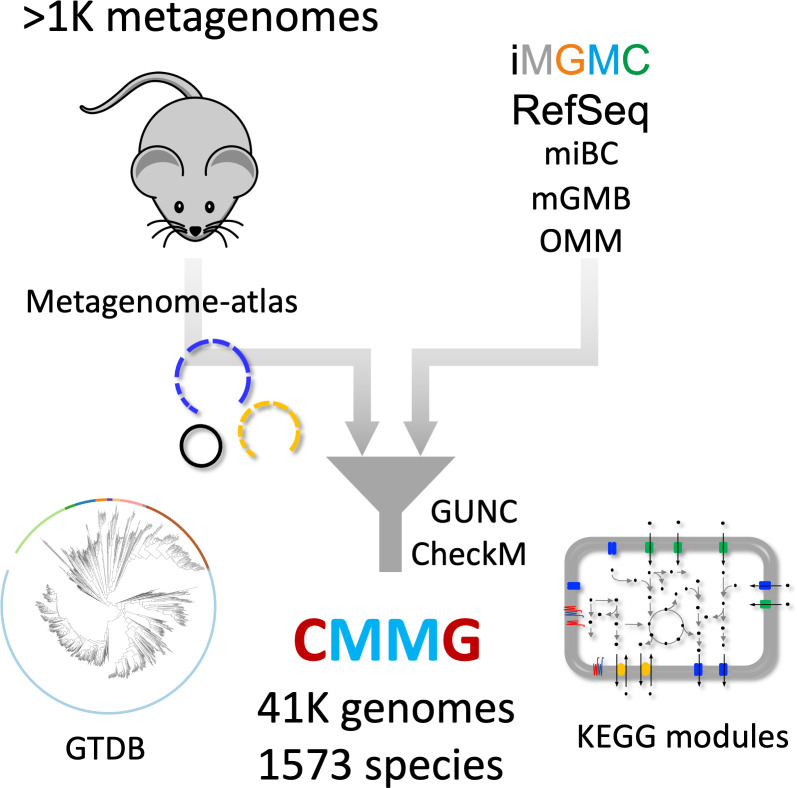
Scheme of the CMMG creation. | iMGMC: integrated mouse gut metagenomic catalog (660 genomes) [10]. miBC: Intestinal Bacterial Collection (53 genomes) [13]. mGMB: Mouse Gut Microbial Biobank (41 genomes) [12]. OMM: Oligo-mouse-microbiota (12 genomes) [11] GTDB: Genome Taxonomy Database [21].

We selected all mouse-associated bacterial genomes retrieved from RefSeq (Fig 1 and S1 Table), incorporating genomes from mouse-specific culture collections [11–13]. We retrieved all metagenomic datasets associated with the mouse intestinal tract sequenced as paired-ends from the NCBI sequence read archive. Together with 92 samples from our lab, this amounted to 1061 samples (S2 Table). Each sample was processed using metagenome-atlas [14], which handles pre-processing, assembly, and binning of the metagenome datasets.

For 60% of the reference genomes, we recovered MAGs that align to them with high coverage and identity (average nucleotide identity (ANI) >95%, IQR 94–99%, S1 Fig). This result validates our metagenome assembly approach to recover “reference quality” genomes *de novo*. Some of the minor differences are likely attributed to strain variation, as the coverage was higher for more similar genomes (S1 Fig).

All genomes were filtered based on fragmentation (N50 >5000), chimerism using GUNC [15], and completeness and contamination were estimated with checkM [16]. Surprisingly, some reference genomes had contamination values of 100%, suggesting that the sequenced genomes consist of multiple strains. In total, we included 771 reference genomes from 249 species to the CMMG catalog (S2A and S2B Fig), while 58 reference genomes did not pass the quality filtering. From the genomes assembled with Metagenome-atlas, 24’708 passed the quality filtering from which one third had high quality (Completeness– 5× Contamination > 90%). We included the MAG catalogs iMGMC and mMAGs [10], of which 75% passed our quality filtering (S2A Fig), resulting in 41’798 genomes (S3 Table). From these genomes, one-third had high quality. The quality metrics and genome contiguity of the high-quality MAGs were comparable to the values of the references (S3 Fig).

Since we assembled genomes from individual samples, the same strain could have been recovered multiple times, especially because different gut locations of the same mouse were sampled. To remove this redundancy, we clustered the genomes based on the ANI calculated using bindash [17]. 95% ANI was used as a threshold to delineate genomes from the same species [18,19]. The species representatives were annotated with the metabolic modules and the genome taxonomy database (GTDB [20,21]). For unclassified species, we manually curated the taxonomy based on phylogenetic placement (S4 Table).

### CMMG species comprehensively cover the mouse gut metagenome

**Fig 2:**
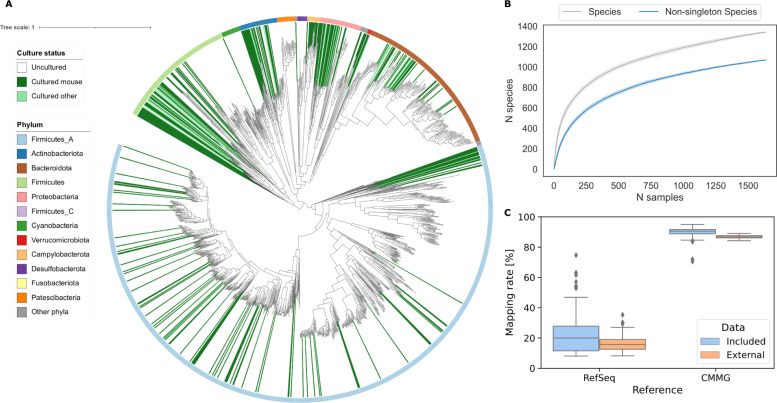
CMMG comprehensively covers the mouse metagenome. **|** (A) Maximum-likelihood phylogenetic tree of the 1’573 bacterial species detected in the mouse gut. Clades are colored by culture status, and the color ring indicates the phylum. (B) Rarefaction curves of species. (C) Comparison of mapping rates of the mouse gut metagenome.

The CMMG collection represents 1’573 species, of which 20% are newly discovered (Fig 2A). We defined 180 new genera and eight new families. 82% of the CMMG species are uncultured, with only 16% having a mouse-specific cultured strain. 152 species do not have a cultured species even at the order level. The sum of cultured species accounts on average for less than 37% of the mouse metagenome.

Rarefaction analysis shows that the number of species approaches a saturation point when considering species with at least two conspecific genomes, indicating that the CMMG catalog contains most of the species commonly living in the mouse gut (Fig 2B). More rare species can still be discovered, as indicated by the non-converging rarefaction curve with singletons (species recovered in one only sample). Based on the curated taxonomy, we generated a Kraken2 [22] database that is consistent for all ranks. Kraken2 achieves a mapping rate of the mouse metagenome of 90.3% using the CMMG, a 4.5-fold increase compared to the standard Kraken database containing all RefSeq genomes from archaea, bacteria, viruses, and plasmids (Fig 2C). To independently evaluate the mapping rate of the CMMG catalog, we used an external dataset of cecum samples, which was explicitly left out from this catalog. The CMMG species covered 85.9% of the metagenomic reads, representing an over 5.4-fold increase to the RefSeq database (Fig 2C). We next compared our catalog to the one previously published by Lesker *et al*. CMMG contains 25% (316) newly-identified species, triples the count of high-quality genomes and consequently the number of high-quality species representatives from 479 to 814 (S2A–S2C Fig), and improves the mapping rage by 6% (S2D Fig). These efforts improve the overall diversity and quality of available species from the mouse gut (S2E Fig).

### CMMG enables comparative analysis of mouse metagenomes by relating functional changes to driver species

To illustrate how this catalog allows discovering compelling biological insights, we analyzed the metagenome from mice exposed to cold ambient temperatures. Cold exposure is a stimulus that activates the brown fat and promotes beige adipose tissue development within the subcutaneous white adipose tissue [23–25]. As such, it is an extensively used intervention for enhancing thermogenic and mitochondrial activity in adipose tissues, leading to decreased adipose tissue amount and improved glycemic status.

**Fig 3:**
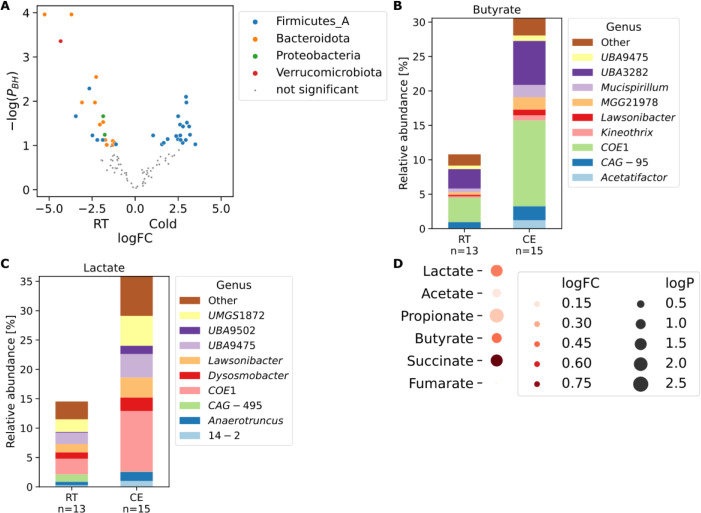
CMMG links functional changes to driver species. **|** (A) Volcano plot of species changes in mouse cecal microbiota upon cold exposure. Significantly changed species are colored by their phylum. P_BH_: P-value corrected for multiple testing using the Benjamini-Hochberg procedure. (B, C), Bar plots of butyrate (B) and lactate (C) in mouse cecal microbiota upon cold exposure. Contribution to the relative abundance of each module is partitioned by genus. (D) Dot-plot of metabolite changes in ceca from germ-free mice transplanted with cold-adapted microbiota compared to RT-microbiota transplanted controls. Source: [26]. CE: Cold exposure, RT: Room temperature control.

We [26], and others [27] showed that cold exposure leads to a marked shift of the microbiota composition observed by 16S analysis, which is in itself sufficient to improve the insulin sensitivity, induce tolerance to cold, increase the energy expenditure and lower the fat content–an effect in part mediated by activation of the brown fat [26,27] and browning of the white fat depots in the cold microbiota-transplanted mice [26,28–31]. These results indicate an existence of a microbiota-fat signaling axis [32,33]; however, the signaling cascades mediating this process remain poorly understood. Therefore, we sequenced the metagenome from feces, and cecum, of the mice from Chevalier et al. 2015 [26], that were cold exposed at 4°C for 30 days, together with their room temperature controls. As noticed previously [26], we confirmed that *Akkermansia muciniphila*, the only representative of the phylum *Verrucomicrobiota* was eliminated by cold exposure (Figs 3A and S4). The most abundant species from the phylum *Actinobacteriota* (*NM07-P-09 sp004793665*) and three *Muribaculaceae* species were even more significantly decreased (P_BH_ < 1e-4, Fig 3A). Cold exposure also led to an increase of the family *Lachnospiraceae* and a decrease in *Muribaculaceae* and *Oscillospiraceae*.

On a functional level, cold exposure led to a doubling of butyrate and lactate production. These changes were mainly due to the increase of the family *Lachnospiraceae*, specifically the uncultured genus *COE1* (Fig 3B and 3C). To address whether these uncovered metagenomic changes are indeed reflected in differences of the actual metabolite levels, we looked at the germ-free mice transplanted with microbiota from the cold-exposed mice or their room temperature (RT)-kept controls. Transplantation of the cold-adapted microbiota indeed caused an increase in the production of butyrate, lactate, propionate, and succinate in the cold microbiota recipients’ cecum compared to those from the germ-free mice inoculated with microbiota from control RT-kept mice (Fig 3D) [26]. Interestingly, the increased lactate was also measured in the cecum and serum of mice with an intermittent fasting feeding regime [34], which was shown to induce browning via induction of the Vascular endothelial growth factor [35]. Similarly, succinate is linked to increased thermogenesis [36]. We found a decrease of the prokaryotic succinate dehydrogenase (S5 Fig), which metabolizes succinate to fumarate, suggesting a mechanistic link between the cold-induced microbiota changes and the adipose tissue browning. We also observed a decrease in Lipopolysaccharide (LPS) synthesis (S5 Fig), both in an LpxL-LpxM–dependent and -independent way, primarily attributed to the cold-induced reduction of *Muribaculaceae*. LPS administration causes reduced core body temperature and heat release, correlated with mitochondrial dysfunction [37]. In contrast, genetic deletion of the LPS receptor, the toll-like receptor 4 (TLR4), confers to resistance against high caloric diet-induced obesity, improves glucose tolerance and insulin sensitivity, and promotes adipose tissue browning [38]. These findings suggest an additional possible link between the cold-induced microbiota changes and adipose tissues both at mechanistic and bacterial level, contributing to improved insulin sensitivity and adipose tissue browning.

This example illustrates the CMMG catalog’s usability as a reference for metagenomic studies, enabling discoveries of precise and comprehensive changes of species and related functions that are induced by a treatment or a disease. The CMMG sets the ground for reanalysis of the existing datasets for uncovering species and bacterial functions that are involved or altered by the condition of interest.

### Comparison between human and mouse gut microbiomes

Studying mouse microbiota and its impact on the host as a proxy for humans implies their similarities. However, 16S rDNA profiling and gene catalogs do not allow a comprehensive analysis of the analogy between human and mouse microbiota down to species level. Also, much fewer species from the mouse gut are sequenced than from the human gut [39]. The CMMG catalog, together with the recent creation of genome collections from the human gut [40], renders this comparison possible. We, therefore, compared the species from CMMG to the ones from the unified human gastrointestinal genomes (UHGG) [40] and applied the same criteria as for species delineation (ANI > 95%). We annotated all species from both hosts with the genome taxonomy database (GTDB, release 06-RS202) and curated the unannotated taxonomic levels to allow a consistent taxonomic comparison from domain down to species level.

**Fig 4:**
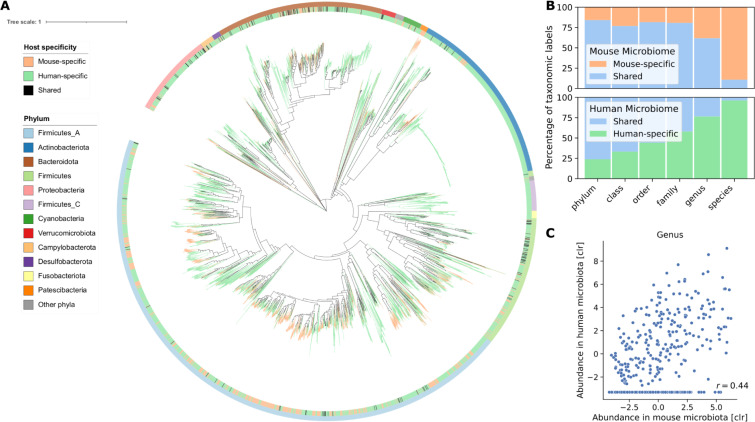
Human and mouse guts harbor distinct bacterial species. **|** (A) Maximum-likelihood phylogenetic tree of the bacterial species from UHGG and CMMG. The innermost color ring and the tree branches are colored by host specificity. The outermost color ring indicates the phylum attribution. (B) Percentage of shared and host-specific taxa from CMMG and UHGG at different taxonomic levels. (C) Correlation of average abundance of genera in human and mice microbiotas. For genera detected in mouse but not in human we imputed the lowest value measured. CLR = centered log ratio.

More than half of the species in both microbiomes belonged to the phyla *Firmicutes_A* (Fig 4A). *Firmicutes_A* and *Bacteroidota* (*Bacteroidetes*) were the most abundant phyla in both human and mouse microbiomes (S6A Fig). Surprisingly, the phylum, *Firmicutes_B* is increased in mice compared to human, and the phylum *Firmicutes_C* is highly underrepresented. Overall, 16 phyla had representatives in both human and mouse microbiome and 5 were only found in human and not in mice. In contrast, the phyla *Deferribacterota*, *Thermotogota*, and the two species *Chlamydia muridarum* and *Chlamydophila psittaci*, which represent an own phylum, were specific to mice. No archaea were reconstructed from the mouse gut metagenome, whereas 0.4% of the genomes in the human gut from the UHGG belonged to this domain. At the family level, we found that humans and mice share 88 of the 109 taxa (80% overlap, Fig 4B), whose average abundance in human and mouse microbiota were strongly correlated (r = 0.75). The two families, *Lachnospiraceae* and *Oscillospiraceae*, dominating *Firmicutes_A*, had high abundance in both human and mice (S6B Fig). The family *Muribaculaceae* was over 30 times more abundant in mice than in humans, whereas *Bacteroidaceae* was 14 times less. While at the genus level, 255 of 412 taxa were shared (62% overlap, Fig 4B), the abundance of the genera showed a moderate correlation (Fig 4C, r = 0.44), in line with results based on 16S rDNA sequencing [41]. Intriguingly, the genus *Collinsella* (phylum *Actinobacteria*), associated with atherosclerosis and rheumatoid arthritis [42,43], was represented with 579 species in the human but not found in the mouse metagenome.

Strikingly, from the 1’573 CMMG species, only 170 (10.8%) were identified in the human gut microbiota (Fig 4A and 4B). The shared species accounted, on average, for 13% of the mouse gut microbiome composition (S7A Fig). Mapping mouse metagenome samples to a human reference database, and vice versa, achieved only 30% mapping rate (S7B Fig). These findings show major differences between human and mouse microbiota at the species level, demonstrating that mice and human microbiota are largely distinct (Figs 4 and S8). These results effectively challenge our view on the analogy between human and mouse microbiota. They may impact the experimental designs, analyses, and approaches for studying the human gut microbiome by using mouse as a proxy.

## Discussion

We generated a comprehensive catalog of the mouse gut metagenome containing 41’798 genomes from 1’573 species. This resource enables mapping of over 90% of the mouse metagenome. Three-quarters of the species are uncultured. Some do not even have a representative at the order level, pointing to the CMMG catalog as a basis for targeted culturing of these missing strains.

CMMG is built by assembling all publicly available data from the most used mouse strains, thus comprehensively representing the microbiome of laboratory mice. Saturation in the rarefaction analysis shows that the CMMG catalog contains most species commonly living in the mouse gut. Nevertheless, we cannot exclude that new sequencing samples may add diversity that is not part of the CMMG, for example, species present in wild mice. Comparing the mouse microbiota to its human counterpart reveals overlap and correlation of the average abundance from phylum down to the family level. As suggested by amplicon sequencing [41], the genera are qualitatively approximately the same but quantitatively rather different. We observed only a moderate correlation between their average abundance in human and mouse microbiota. Whereas a comprehensive and precise comparison at species level between the two microbiomes was not previously feasible [1,39], the comparison of CMMG with the UHGG collection reveals an overlap of only 10% of the species. In part, these findings are in agreement with the recently published Mouse Gastrointestinal Bacteria Catalogue [44], which contains 1,094 species, and shows an overlap of only 3% at the species level between human and mouse.

While the overlap at the genus and higher taxonomic levels may imply a functional similarity of the human and mouse microbiome, this assumes that functions are conserved within a taxon. While this is indeed the case for some functions, the functional annotation is biased towards more conserved functional annotations, which can be transferred from model organisms to less-studied bacterial species. Species from the same genus, and even strains from the same species, can have divergent functions. Strains from the same species can differ in up to 30% in their gene content [45], which may help strains from the same species to adapt to different environments. This is especially well studied for the species *Limosilactobacillus reuteri*, which has mouse- and human-adapted strains, however, with very different functions [46,47].

Different ways can be envisaged to overcome the challenges imposed by these findings in using mouse microbiota as a model for human. For example, creating ‘humanized’ mouse models by transplantation of human gut microbiota into germ-free mice or complementing the work by exploring additional animal models [48]. To leverage data produced using conventional mice, it will be important to uncover functional homologs between the species adapted to mouse and human microbiota, e.g., by identifying ‘guilds’ [49], groups of species that use the same type of resources in a similar way. The provided consistently functionally annotated species of the human and mouse microbiome lays the basis for such work.

In summary, the knowledge of the genomes and the nearly complete mapping rate provided in CMMG enables uncovering species and bacterial functions that are involved or altered by the condition or treatment of interest. Our resource containing a comprehensive collection of the species from the mouse gut and their functional capacity sets the ground for thorough reanalysis of the existing datasets. It allows analysis of the mouse gut microbiome at an unprecedented depth.

## Methods

### Sequencing of metagenomic data of mice

Animals were on C57Bl/6J background, commercially available through Charles River, France. The cold exposure experiment in mice is detailed in [26]. Paired-end metagenomic libraries were prepared from 100 ng DNA using TruSeq Nano DNA Library Prep Kit (Illumina) and size selected at about 350 bp. The pooled indexed library was sequenced using a HiSeq4000 instrument at the iGE3 facility at the University of Geneva. 15 Cecum and 13 Fecal samples had good quality for analysis.

### Collection of public genome and metagenome data

We queried the sequence read archive (SRA, accessed December 2019) of the National Center for Biotechnology Information (NCBI) for all publicly available paired-end metagenome runs from the mouse microbiome. We specifically excluded samples from human origin and amplicon sequences and other body parts than the gut. We extracted 1061 metagenome samples belonging to 40 projects. Metadata was retrieved using Bio Services [50] and curated (S1 Table). We retrieved 776 assemblies from Ref Seq linked to a biosample collected from mice (S1 Table). We excluded reference genomes collected from other body parts than the gut or feces. The genomes retrieved from Ref Seq, which also incorporates genomes from mouse specific culture collections: Oligo-mouse-microbiota [11] (12 genomes), and Mouse Gut Microbial Biobank (mGMB, 41 genomes) [12], belonged to 279 species (S1 Table, S1 Fig). As genomes of the mouse Intestinal Bacterial Collection (miBC, 53 genomes) [13] were not available, we assembled them from the raw reads.

### Metagenome assembly and binning

Metagenomics and genomic reads were processed using the metagenome-atlas v2.3 [14] pipeline with the command ‘atlas run genomes’. In short, using tools from the BBmap suite v37.78, reads were quality trimmed, and contaminations from the mouse genome were filtered out. Reads were error corrected and merged before being assembled with metaSpades v3.13 [51]. Contigs were binned using metabat2 v 2.14 [52] and maxbin2 v2.2 [53], and their predictions were combined using DAS Tool v 1.1.1 [54]. For assembling the 53 genomes of the mouse intestinal bacterial collection, we used the assembly workflow of metagenome-atlas and set ‘spades_preset: normal’, which uses the Spades as assembler [55]. The quality of the genomes was estimated using checkM v1.1 [16].

### Genome filtering and species clustering

#### Code is available from: https://github.com/SilasK/FastDrep

All genomes were filtered based on fragmentation (N50 >5000) and a quality score was calculated from the output of checkM [16] as ‘completeness minus 5 times contamination’. Aligned with the MIMAG-criteria [56] and other genome catalogs [40], bins with a quality score of <50 were excluded, and genomes with a quality score >90 were counted as high quality or ‘near complete’. Genomes with good quality were grouped into species with average nucleotide identity (ANI) > 95%. For this, all pair-wise average nucleotide identities (ANI) above 0.8 were estimated using bindash [17]. The genomes were pre-clustered into clusters that contain at least one pair of genomes above the threshold. Then each cluster was grouped into species by hierarchical clustering with average linkage using scipy [57]. As for the UHGG [40], the genome with the highest score was selected as the representative for each species cluster based on the following formula:

Score=Qualityscore+0.5×log(N50)+100×isIsolate

Where *Quality score* is the score mentioned above used to filter genomes, *N50* is the N50 score of the assembly contiguity, and *isIsolate* is 1 for isolates and 0 for MAGs, to ensure that isolated genomes are preferred over MAGs even if they have a lower quality score.

### Phylogenetic and taxonomic analysis

The species representatives of both the CMMG and the unified human gastrointestinal genomes (UHGG) [40] were annotated using the genomic taxonomy database toolkit (GTDB-tk v1.2 [20]) and the GTDB release 06-RS202. A maximum-likelihood tree for the CMMG alone and the CMMG combined with the UHGG based on the 120 bacterial marker genes from the GTDB was built using Fasttree v2.1 [58] and rooted at the midpoint. The phylogenetic trees are visualized with iTOL v5 [59]. Genomes defined as new taxa based on relative evolutionary divergence (RED) with GTDB-tk were manually annotated as defining genera and families at comparable RED values as annotated sister clades.

### Inferring cultured status

Species that contain a reference genome from a culture collection included in the CMMG catalog were counted as cultured from a mouse origin. If GTDB-tk [20] annotate the species to a reference with ANI >95%, and the GTDB-tk type species was marked as cultured, we counted the species as cultured from a non-murine source. In both cases, if the reference genome was excluded from RefSeq (i.e., metagenome-assembled genomes) or labeled as uncultured, we counted the species as isolated but not cultured.

### Quantification

Based on our curated taxonomy, we build Kraken 2 and bracken [22] databases for the CMMG and the UHGG using FlexTaxD [60] with a snakemake pipeline available from: https://github.com/SilasK/Kraken. For benchmarking the mapping rate, we used the 184 fecal samples from the MGC v1 [3], which were included in CMMG and mMAG. The dataset from [61] served as an independent benchmarking set. The mapping rates were calculated as the reads attributed with bracken at the species level divided by the the total reads. For comparison, we quantified reads using the standard Kraken2 database accessible from https://benlangmead.github.io/aws-indexes/k2 (as of December 2020). For most quantifications, the mapped reads per genome were summed, and the centered log-ratio (CLR) was calculated using the sci-kit bio package (http://scikit-bio.org/) after imputing zeros using a multiplicative replacement approach. The replacement uses, by default, a delta of 1/*N*^2^, where *N* is the number of species. To calculate the average species abundance in the mouse and human metagenome, we used 1319 samples from the mouse metagenome and a random subset of 1000 samples of the human metagenome that is commonly used for benchmarking [8]. The Pearson correlation between the abundance of taxonomic groups in the human and mouse microbiota was performed with scipy v1.4.1 [57]. For mapping reads directly to genomes, we used BBsplit (https://jgi.doe.gov/data-and-tools/bbtools/bb-tools-user-guide/) with the parameters’ ambiguous2 = best minid = 0.9’ to map the metagenomic reads to the references with 90% identity. We estimated the genome coverage as the median of coverage over 1000bp blocks.

### Functional annotation

The species representatives of both the CMMG and the UHGG were annotated using DRAM [62]. A Kegg-module was inferred to be present if ¾ of all the steps were present in a genome. As Kegg has no modules for short-chain fatty acids, we created custom modules (see the ‘Code’ section). The step coverage was calculated with DRAM for all Kegg modules. The metagenome-side abundance of functional modules was calculated as a sum of the relative abundances of all genomes containing a module. We used the Welch test and Benjamini-Hochberg correction to estimate the significance of changes in module abundance between experimental groups.

### Declarations

#### Ethics approval

The experiments in mice used for sample collection and metagenomic sequencing were approved by the Swiss federal and Geneva cantonal authorities for animal experimentation (Office Vétérinaire Fédéral and Commission Cantonale pour les Expériences sur les animaux de Genève).

## Supporting information

S1 Fig Assembly recovers reference genome with high coverage. Density plot of the coverage vs. identity of the MAGs alignments to 494 reference genomes.
(EPS)

S2 Fig Comparison of sources for CMMG. (A) Overlap of the species from the sources used to generate CMMG. (B) Number of genomes passing quality filtering from this study, Lesker *et al*. and RefSeq. (C) Bar plot showing the top quality score for each specis in CMMG and Lesker et al. (D) Mapping rate for mMAG [10] and CMMG for a mouse fecal dataset included in both catalogs and a dataset not included in both datasets. (E) Rarefaction curve of number of species recovered for increasing number of samples from the different sources and CMMG as a whole. Genomes from iMGM were excluded as they originate from a co-assembly. iMGMC: integrated mouse gut metagenomic catalog [10] mMAG: mouse MAGs [10]
(EPS)

S3 Fig Metagenome-assembled genomes have comparable quality to reference genomes. Violin plots showing the quality score, completeness, contamination estimated using checkM, and the log_10_ N50 from the assembly for the reference genomes and MAGs present in CMMG.
(EPS)

S4 Fig Compositional changes in cold-adapted microbiota. (A) Bar chart of microbiota composition summarized at family level. (B) PCA of CLR-transformed species abundance of microbiota from cold-adapted mice versus their RT-kept controls. CE: cold exposed RT: Room temperature
(EPS)

S5 Fig Metabolic map of Kegg modules changed upon cold exposure. Kegg modules with significantly changed abundance in the cecal microbiome of upon cold exposed mice. Blue indicates decreased pathways; red, increased. The thickness is a function of the *P*-value.
(EPS)

S6 Fig Human and mouse microbiome are similar at higher taxonomic levels. Distributions of abundance of all phyla (A), and most abundant families (B), in the human and mouse microbiome.
(EPS)

S7 Fig Human and mouse guts harboures different sets of species. (A) Relative abundance of mouse-specific taxa and taxa shared with the human at different taxonomic levels. (B) Cross-mapping between human and mouse microbiomes samples and human and mouse-specific databases (CMMG and UHGG).
(EPS)

S8 Fig Fraction of mouse species that are shared with human by phylum. Heatmap of the fraction of mouse species that are shared with the human microbiome at different taxonomic levels grouped by phylum.
(EPS)

S1 Table Reference genomes associated with the mouse gut. The table lists the assembly information of reference genomes associated with the mouse gut. These genomes were filtered for completeness and contamination before integration into CMMG. The columns `Isolated`and `Cultured`label if the genome is Isolated and cultured. The `collection`describes if the genome is part of a mouse-specific culture collection. The genomes of the miBC collection are assembled for this study.
(XLSM)

S2 Table Metagenome samples used to construct CMMG. The table contains the metagenome samples used for the generation of CMMG. The CMMG_Id corresponds to the SRA read id, except for the samples sequenced by our lab. The table contains information retrieved from NCBI that was available for most of the samples: Name, description, Link to bioproject, collection data, country, and submission center. The column ‘Source’ specifies the organ from which the sample was taken. The column ‘Mouse strain’ indicates the strain specification of the host. If the information was available in any of the metadata. Samples of the cold-adapted microbiota under the bioproject accession PRJNA646351 were sequenced for this study.
(XLSM)

S3 Table Genome information for all genomes in CMMG. The table provides the quality metrics, calculated using CheckM, information if the genome is an isolate, and Genome size metrics (Length, number of contics/scaffolds, N50-value), for each genome in CMMG. It contains links to the source (Original bin name, Sample and BioSample of the origin, and Source dataset). The connection to the species is possible via the genome id of the species representative.
(XLSM)

S4 Table Curated taxonomy for all bacterial species from mouse and human. The Table Shows manually curated taxonomy based on GTDB for all species from UHGG and CMMG.
(XLSM)
